# A Review on Impedimetric and Voltammetric Analysis Based on Polypyrrole Conducting Polymers for Electrochemical Sensing Applications

**DOI:** 10.3390/polym13162728

**Published:** 2021-08-15

**Authors:** Nurul Akmaliah Dzulkurnain, Marliyana Mokhtar, Jahwarhar Izuan Abdul Rashid, Victor Feizal Knight, Wan Md Zin Wan Yunus, Keat Khim Ong, Noor Azilah Mohd Kasim, Siti Aminah Mohd Noor

**Affiliations:** 1 Department of Chemistry and Biology, Centre for Defence Foundation Studies, National Defence University of Malaysia, Sungai Besi Camp, Kuala Lumpur 57000, Malaysia; nurulakmaliah86@gmail.com (N.A.D.); jahwarhar@upnm.edu.my (J.I.A.R.); 2 Research Centre for Chemical Defence, National Defence University of Malaysia, Sungai Besi Camp, Kuala Lumpur 57000, Malaysia; marliyanamokh@yahoo.com (M.M.); victor.feizal@upnm.edu.my (V.F.K.); ongkhim@upnm.edu.my (K.K.O.); azilah@upnm.edu.my (N.A.M.K.); 3 Centre for Tropicalisation, National Defence University of Malaysia, Sungai Besi Camp, Kuala Lumpur 57000, Malaysia; wanmdzin@upnm.edu.my

**Keywords:** electrochemical sensor, biosensor, impedimetric, voltammetric, polypyrrole, conducting polymer

## Abstract

Conducting polymers have been widely used in electrochemical sensors as receptors of the sensing signal’s analytes and transducers. Polypyrrole (PPy) conducting polymers are highlighted due to their good electrical conductive properties, ease in preparation, and flexibility of surface characteristics. The objective of this review paper is to discuss the theoretical background of the two main types of electrochemical detection: impedimetric and voltammetric analysis. It also reviews the application and results obtained from these two electrochemical detections when utilizing PPy as a based sensing material in electrochemical sensor. Finally, related aspects in electrochemical sensor construction using PPy will also be discussed. It is anticipated that this review will provide researchers, especially those without an electrochemical analysis background, with an easy-to-understand summary of the concepts and technologies used in electrochemical sensor research, particularly those interested in utilizing PPy as a based sensing material.

## 1. Introduction

**Figure 1:**
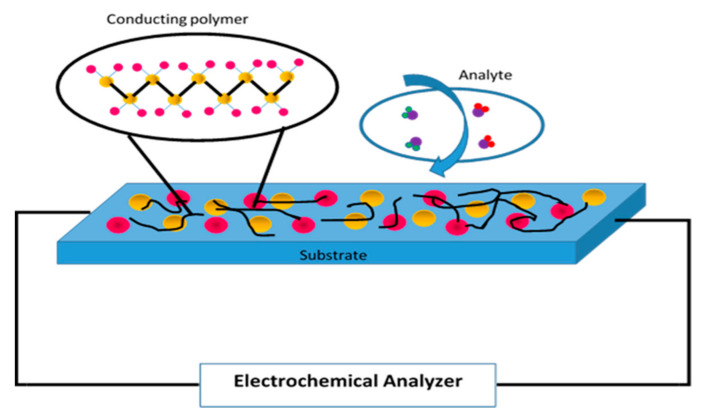
Schematic diagram of a conducting polymer and analyte on an electrochemical sensor.

Conducting polymers (CPs) play an important role in the design and development of electrochemical sensors. They provide the necessary electrical conductivity to transduce the occurrence of the coupling event into an analytical signal (Figure 1) [1].

CPs are conjugated polymers that possess delocalized π-bonds on the backbone of the polymers. These π-bonds assist in the migration of electrons throughout the polymeric chain. Thus, these polymers can act as conductors, semiconductors, or even superconductors. Besides that, CPs also have high electron affinity and redox activity. The general physical properties of CPs depend on the size and length of the conducting polymer, which can also be described in terms of their molecular weight [2]. There are many types of CPs such as polyaniline (PANI), polypyrrole (PPy), polyacetylene, polyparaphenylene (PPPh), polyparaphenylene vinylene (PPV), polythiophenes (PTh), polydiaminonaphthalene, polyazulene, and poly (3,4-ethylene dioxythiophene) (PEDOT) [3]. Electrically conductive polymers are a class of materials that can be fabricated to generate either transient or static electrical charges because of their physico-chemical properties [4]. Among those polymers, PPy is the most extensively used as a conducting polymer in electrochemical sensing applications because it is a heterocyclic conducting polymer, with good environmental stability, high electronic conductivity, and biocompatibility compared to other CPs [5,6,7,8].

Furthermore, PPy is easy to synthesize as a black powder chemically and electrochemically as thin films on various electrodes for both aqueous and nonaqueous media [4,9,10,11]. PPy has also been applied in multidisciplinary applications such as batteries, supercapacitors, coatings, etc. [12,13,14]. Despite these excellent properties, it also suffers from certain drawbacks, namely, poor thermal solubility and low mechanical stability [15]. These drawbacks can affect the response characteristics and sensitivity to small perturbation of the electrochemical sensor [3]. In the past two decades, scientists have applied different approaches to modifying PPy to improve its properties. These approaches include blending [16], electro-polymerization [17], interpenetrating network formation, and composite synthesis [18,19]. 

### 1.1. Synthesis and Preparation of Polypyrrole

PPy is commonly synthesized through chemical or electrochemical oxidation of pyrrole monomer using oxidant agents through a conjugated bond system with the polymer backbone. However, in the presence of only PPy, it may suffer from a certain drawback as mentioned above, and this will restrict the device’s application. Therefore, approaches such as blending, electro-polymerization, interpenetrating network formation, and composite synthesis have been done to enhance the PPy properties, thus improving the device’s performance.

Hosseini and Entezami, 2003, prepared the polymer by blending PPy with polyvinyl acetate (PVAc), polystyrene (PS), and polyvinyl chloride (PVC) using a chemical method to produce flexible and free-standing blended polymer films. The sensing abilities of these films towards toxic gases and vapors were investigated and it was discovered that the PPy blended film had improved mechanical strength and was also able to exhibit greater environmental stability. Besides that, it was also found the sensoric properties of the PPy blends towards toxic gases and vapors against hydrogen halides, hydrogen cyanide, and halomethyl compounds were better than non-blended PPys. Therefore, it can be surmised from their findings that PPy blends are good candidates for sensing toxic gases and vapors [16].

Meanwhile, Song et al., 2019, prepared three-dimensional graphene oxide with an interconnected porous polypyrrole (pGO/PPy) nanostructure-based actuator through electro-polymerization and sonication. This configuration allowed the actuator to adsorb trace cadmium by the carboxyl functional group in the GO and also was able to widen the electrode detection range of the PPy which was densely covered with gold substrate. From the results obtained, they suggest that the pGO/PPy was a promising material that had an ability to enhance the pre-concentration factors, enrich the potential window and greatly increase the sensitivity of the cadmium sensor. The cadmium detection in the presence of interference ions showed good selectivity using this pGO/PPy nanostructure based actuator. Besides which, the nanostructure also achieved a wider linear range and a lower limit of detection (LOD). Moreover, this method could be developed into a low cost, portable and reliable sensor that is both sensitive and selective towards cadmium in aqueous systems and could potentially facilitate detection of other heavy metals such as lead, mercury and copper [17].

Hassanein et al., 2017, fabricated biosensors based on chitosan-ZnO/Polypyrrole nanocomposites. The sensor was prepared using the oxidative polymerization of pyrrole monomer with (NH_4_)_2_S_2_O_8_ as the oxidant and followed by mixing with chitosan–zinc oxide composites. The conductive polymers and oxide nanoparticles (organic–inorganic nanocomposite materials) have been previously widely used because of the novel properties of this nanocomposite which can be attributed to the successful blending of the individual characteristics of the parent constituents into a single material. The advantages of the oxide nanoparticles are in their ability to modify their chemical, mechanical, electrical, structural, morphological, and optical properties under specific circumstances. Moreover, these nanostructure materials have a larger percentage of surface atoms available which possess high reactivity. From the results, it was found that there was a significant improvement in electrical conductivity from the cyclic voltammetry measurements of the K_3_(Fe(CN)_6_) sample. A large enhancement of the stripping of peak current compared to bare CPE was identified using the square-wave adsorptive anodic stripping voltammetry method. Consequently, the proposed material proved to possess suitable ability as sensing materials in biosensor applications [18].

In other work done by Tlili et al., 2005, they reported the technique of interpenetrating network formation, where they immobilized DNA probes bearing amine groups by covalent grafting on a supporting polypyrrole matrix functionalized with activated ester groups. The immobilization step played an important role in determining the overall performance of the biosensor. In order to achieve high sensitivity and selectivity, it required minimization of non-specific adsorption and stability of the immobilization. Polypyrrole (PPy) conducting polymer was chosen in their study because of its biocompatibility, high hydrophilic character combined with high stability in water and facile incorporation of many counter ions which make it highly suitable as an interface for grafting DNA probes onto a micro-sized surface. From the results, it was discovered that the large surface area obtained by using porous polypyrrole leads to an increase in the density of the immobilized DNA probes, which then helps to monitor more easily the DNA hybridization reaction [19]. 

In yet another study, Hsu et al., 2014, used the electropolymerization method to incorporate chloro(protoporphyrinato) iron(III) (hemin), polypyrrole (PPy), and silver (Ag) in order to achieve sufficient sensitivity for an environmental dissolved oxygen (DO) sensor. The electropolymerization method provides a strong adhesive bond at the substrate/hemin interface and allows for an increased concentration of hemin. However, due to their poor current collection capacity, electropolymerized films with higher hemin loading do not instead produce proportionally higher current or increased sensitivity. Therefore, co-electropolymerizing hemin and pyrrole to fabricate a sensing electrode for dissolved oxygen sensing applications is one of the better methods for solving this lower sensitivity problem. Thus, this sensor is able to be manufactured at a lower cost and with longer lifespan. In addition, since it is a solid state sensor, it has the potential to be miniaturized and integrated within a micro-fabricated reference electrode to form a complete sensing system at a very low cost [20].

### 1.2. Electrochemical Sensors

Electrochemical sensors have aroused great interest in providing fast and highly sensitive detection of proteins, which include the antibodies generated during the immune response against infections. Recently, electrochemical sensors have found widespread applications in clinical diagnostics, food safety, food quality, biological analysis, and environmental monitoring. Electrochemical sensors are sensors that change the effect of an electrochemical reaction of target species on the electrodes into useful signals which demonstrate alterations in potential and conductivity. With the utilization of biofunctionalities in the electrochemical sensor such as recognition and catalysis, these sensors are known as “biosensors” [21]. A typical biosensor is a combination of a transducer and certain biological elements. The biosensor can also be described as an electrochemical, optical, thermal, or piezoelectric biosensor depending on the type of transducer used. 

**Figure 2:**
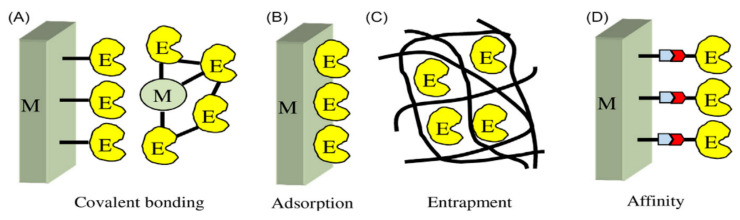
Schematic representation of strategies for enzyme immobilization: Covalent bonding (**A**); Adsorption (**B**); Entrapment (**C**) and Affinity (**D**). E, enzyme; M, matrix [22]. Reproduced with permission from [H. Muguruma], [Biosensors: Enzyme Immobilization Chemistry]; published by [Elsvier], [2018].

In order to functionalize the biological element during the development of the biosensor, the immobilization step is a crucial step. The most popular biosensor utilizes enzymes as the biological substance with an electrode functioning as a transducer. The enzyme is directly immobilized onto the surface of the electrode, or is immobilized onto the electrode using a suitable matrix. In most cases, there are four strategies for enzyme immobilization that can be considered. These strategies, derived from the chemistry of immobilization, are shown in Figure 2. The first strategy is covalent bonding to the electrode or matrix (Figure 2A). The second is physical adsorption to the electrode or matrix (Figure 2B). The third is entrapment involving the enzyme and the electrode (Figure 2C). The final is affinity, which uses a specific biochemical interaction (Figure 2D). However, these strategies cannot each be considered as the perfect strategy because the optimum biosensor still needs to be selected depending on the specific enzyme and transducer [22].

The capture of a target analyte onto a bio or non-bioreceptor immobilized on a CP will generate a measurable analytical signal which is then converted into an electrical signal. The electrical signal can be identified using three main types of sensing modes such as potentiometric (membrane potential change); impedimetric (impedance change); and voltametric or amperometric (change of current for an electrochemical reaction with the applied voltage in the former, or with time at a fixed applied potential in the latter) [23]. It is desirable to strategize the development of an electrochemical sensor in order to provide low detection limits, high selectivity, and utilize a limited amount of indicator species. The impedimetric mode measures the targeted analyte through the output of an electrical impedance signal that is proportional to the analyte activity [9,24]; whereas in the voltametric mode, data about an analyte are obtained by measuring the current while applying a working electrode potential. The electrochemical reaction on the electrode surface and the electrode/electrolyte interface layer produces the final current [25]. The voltametric strategies include linear sweep voltammetry (LSV), cyclic voltammetry (CV), differential pulse voltammetry (DPV), and square wave voltammetry (SWV). These strategies provide a detailed measure with a broad range of effectiveness and a low detection limit. Meanwhile, in the amperometric mode, a consistent potential is connected to the working electrode, resulting in a current over time measurement. The difference between the amperometric and voltametric modes is the use of a potential step instead of a potential sweep. Using the amperometric mode will provide more selectivity and sensitivity since the reduction or oxidation potential utilized in the determination is normal for the analyte sample [26]. 

In this review, the discussion of sensing modes for the electrochemical sensor will be focused on impedimetric and voltametric modes. It will be start with an introduction followed by a description in detail, then the practical impacts of these modes on the electrochemical sensors will be discussed. We aim to present and highlight how these sensing modes can contribute to rationalizing the optimization of PPy based conducting polymers in electrochemical sensor applications. 

## 2. Impedimetric Sensing Mode

In order to describe the response of a PPy based conducting polymer electrochemical sensor towards a low amplitude sinusoidal perturbation as a function of frequency, the impedimetric sensing mode will be used. Impedimetric mode, also known as impedance, is a circuit’s ability to measure the resistance towards the flow of an electrical current. To measure the impedance, an electrochemical impedance spectroscope (EIS) is used with the application of a small sinusoidal potential to the working electrode in an electrochemical cell, while measuring the resulting current response. By varying the excitation frequency, *f*, of the applied potential over a range of frequencies, one can calculate the complex impedance, i.e., the sum of the system’s real and imaginary impedance components as a function of the frequency (i.e., angular frequency *w*). The results of the impedance measurement can be graphically demonstrated using the Nyquist (Cole–Cole) and Bode plot for all the applied frequencies with the real part of the impedance *Z* plotted along the *x*-axis and imaginary part plotted along *y*-axis in the latter [27].

### 2.1. Nyquist Plot 

The Nyquist plot is a plot where the imaginary impedance *Z*”(*ω*) is plotted against real impedance *Z’*(*ω*). Generally, the resistance value can directly obtain from Ohm’s law as shown in Equation (1), where the resistance is the ratio between voltage, *E*, and current, *I*.

(1) $$R=\frac{E}{I}$$

It assumes an ideal resistor. An ideal resistor occurs when it follows the Ohm’s law at all voltage and current levels, where the resistance value is independent of frequency and when AC voltage and current signals are in phase with each other while going through the resistor. However, this does not always happen because in reality, circuit behavior is far more complicated. Therefore, in this concept, the impedance element is much more suitable for use rather than simple resistance to explain the changes measured in the circuit. Impedance is a frequency-dependent measurement of the opposition to current flow in an electric circuit. Impedance measurement is performed by applying an AC excitation voltage to an unknown system while measuring the current. The ratio of the excitation voltage to the current gives the complex impedance of the system. 

The first step after impedance measurement is done is the graphical representation from the experimental data. The data from the impedance measurement will consist of three main components which are the real and imaginary impedance, and the frequency. These data will then be represented in Cartesian coordinates as shown in Equation (2):

(2) $$Z\left(i\omega_{i}\right)=Z_{i}^{\prime }+iZ_{i}^{\prime \prime }$$

Or in polar coordinates as shown in Equation (3):

(3) $$Z\left(i\omega_{i}\right)=\left|Z_{i}\right|e^{i\varphi_{i}}$$

where $\left|Z_{i}\right|={\left(Z_{i}^{\prime 2}+Z_{i}^{\prime \prime 2}\right)}^{1/2}$ is the modulus and $\varphi_{i}=tan^{-1}\left(Z_{i}^{\prime \prime }/Z_{i}^{\prime }\right)$ is the phase, which corresponds to a given frequency. 

**Figure 3:**
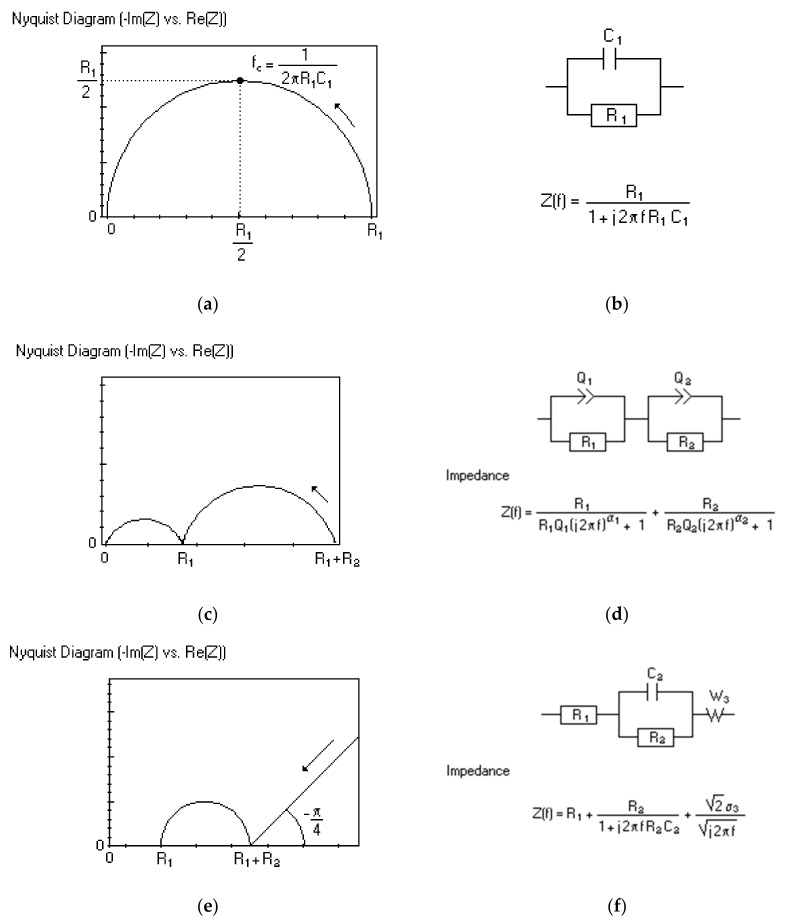

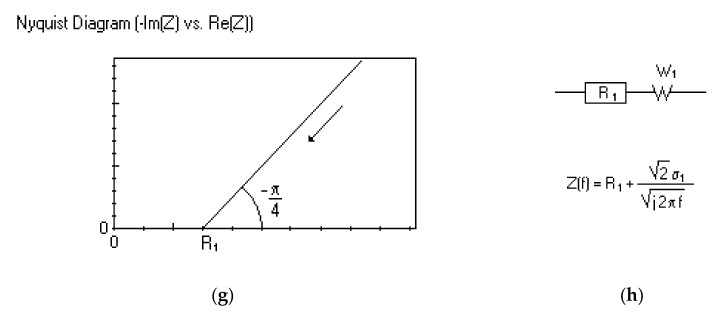
Nyquist plot with one semircircle and its equivalent circuit (**a**,**b**); two semicircles and its equivalent circuit (**c**,**d**); one semircircle with spike (45°) and its equivalent circuit (**e**,**f**); only spike (45°) and its electrical equivalent circuit (**g**,**h**) [28].

The most common plot for impedance representation is based on Equation (2) which is a Nyquist plot with only one semicircle (Figure 3a). It shows the results from an electrical equivalent circuit that is depicted in Figure 3b, which consists of a resistor and a capacitor in parallel. The direction of the frequency scanning is from high to low frequencies. At higher frequencies, the capacitor’s impedance will be very low and a major part of the current will flow through the capacitor. With a decrease in the frequency, the capacitor’s impedance increases and a bigger fraction of the current will then flow through the resistor. When most of the current flows through the resistor, the total imaginary resistance *Z*” will drop as the real part *Z*’ increases. Sometimes, the plot may consist of several semicircles or only a portion of a semicircle (Figure 3c,e). The different semicircles represent different electrical equivalent circuits and are shown in Figure 3d,f.

The equivalent circuit derived from the plot could then be used to analyze changes or the effects on the electrochemical sensor system that was added or modified. Besides that, the charge transfer resistivity, *R*_ct_, also can be obtained from the Nyquist plot. For example, Ramanavicius et al. described an immunosensing system model based on the bovine leukemia virus (BLV) protein (gp51) entrapped within electrochemically synthesized polypyrrole (PPy/gp51). They reported that another element was present in the blood serum sample after it was treated as detected in the fitted equivalent circuit. This was due to the slightly increased measurement of the real part of the impedance spectrum (resistivity increased). It was explained that an additional layer occurred outside the polypyrrole film and proved that the treatment was successful [29]. Devi et al. reported that *R*_ct_ value was increased with the addition of xanthine oxidase (XOD) in a ZnO-NPs/PPy/Pt electrode, which is due to the immobilization of XOD onto the ZnO-NPs/PPy/Pt surface. It proved that the use of nanocomposites and PPy electrodeposited on the Pt surface electrode as a support for the immobilization of XOD resulted in an improvement of the xanthine biosensor performance with a detection limit of 0.8 μM [30]. Meanwhile, Chen et al. used impedance analysis to evaluate the charge separation efficiency of a PPy based photoelectrochemical sensor. There were two semicircles obtained from the impedance curve when Cu_2_O was added on top of the ITO electrode. The semicircles known as Rct were then reduced to one and became smaller when fabricated with and without Microcystin-LR and LiClO_4_ as template molecules during the electropolymerization process. As *R*_ct_ become smaller, the charge transfer efficiency become higher [31]. Furthermore, Bao et al. electrodeposited gold nanoparticles/polypyrrole-reduced graphene oxide nanocomposites (Au/PPy-rGO) on top of a bare glass carbon electrode (GCE) in order to produce excellent sensing performance for mRNA-16. The *R*_ct_ from a small semicircle (bare GCE) was decreased to almost a straight line (after electrodepositing) in the impedance curve results. When it is being further assembled using catalyzed hairpin assembly (CHA), and hybridization chain reaction (HCR), the *R*_ct_ semicircle was greatly increased, demonstrating the successful CHA and HCR processes and the fact that more negatively charged DNA polymers were linked on the modified electrode [32]. Akshaya and co-workers researched a Palladium–Gold (PdAu) based electrochemical sensor which was developed by electrodepositing PdAu nanoparticles onto a Polypyrrole (PPy) modified carbon fiber paper (CFP) electrode. They found that with the modification of CFP using the PPy conducting polymer, the *R*_ct_ decreased, indicating the conducting nature of PPy. Then, with further electrodeposition of PdAu nanoparticles onto the PPy/CFP, the value of *R*_ct_ become significantly decreased. This confirmed the formation of a highly conducting electronic pathway at the electrode–electrolyte interface where Pd and Au nanoparticles facilitated electron transfer between the analyte and the electrode [33].

### 2.2. Bode Plot

**Figure 4:**
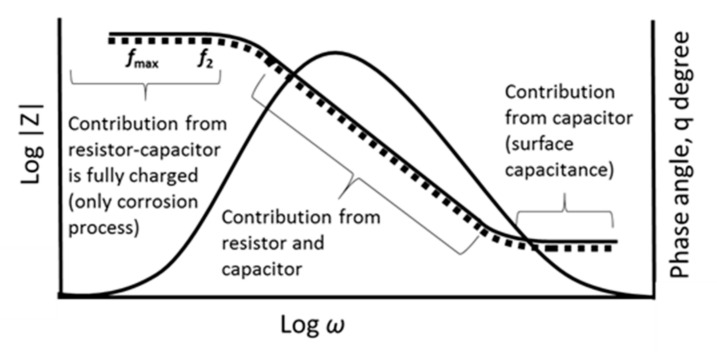
Bode plots of the frequency response (dotted line) and phase angle (solid line) for an electrochemical system.

Even though the Nyquist plot can give significant information on the resistance of the material used, it has one major shortcoming where it is unable to show the frequency used at the focal data point needed. Each point corresponds to a given frequency which is $\omega \;\left(s^{-1}\right)$ or $f\left(Hz\right)$, where ω=2πf. Therefore, as an alternative, the data can be represented in a Bode plot by using Equation (3). Generally, the Bode plot provides a more comprehensible description of the electric systems’ frequency-dependent behavior than the Nyquist plot, in which frequency values are not clear. In the Bode plot, the data are plotted with log of frequency on the *x*-axis and both the log of absolute value of the impedance (|*Z*|) and phase-shift (*θ*) on the *y*-axis (Figure 4) [34].

A typical Bode plot is the same system as shown in Figure 2A. It is simpler to understand as there is only one semicircle that appears on the Nyquist plot. The log |*Z*| versus log *ω* curve can be used to determine the values of *R*_p_ and *R*_Ω_. At very high and very low frequencies, |*Z*| becomes independent of frequency. At the highest frequencies, the ohmic resistance controls the impedance and log (*R*_Ω_) can be read from the high frequency horizontal level. On the other hand, at the lowest frequencies, log (*R*_p_ + *R*_Ω_) can be read from the low frequency horizontal portion [35].

**Figure 5:**
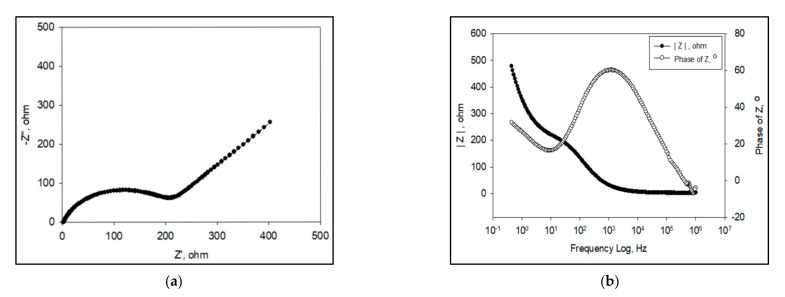
Nyquist plot with two semicircles (**a**) and Bode plot (**b**).

Besides that, the Bode plot can also prove the number of semicircles present in the corresponding Nyquist plot. It can be seen from the shapes of the phase angle plots. For example, from the Nyquist plot, a smaller semicircle appears at higher frequencies, followed by second larger semicircle at medium frequencies and a Warburg diffusion effect in low frequencies (Figure 5a). Therefore, to confirm the presence of these two semicircles, the shape from the phase angle graph in the Bode plot will be used (Figure 5b). It can then be seen that the slope is somewhat broadened. Therefore, it proves that there is more than one semicircle present in the Nyquist plot.

**Figure 6:**
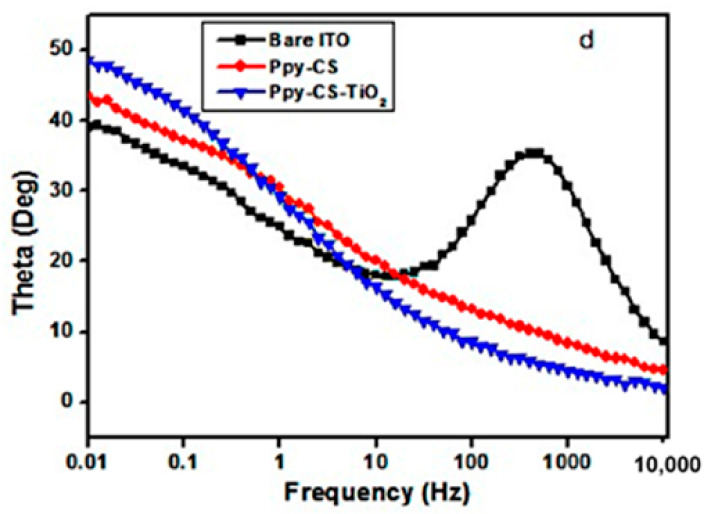
Bode phase plot for 1 mM K_3_(Fe(CN)_6_) in 0.1 M KCl at a scan rate of 50 mV s^−1^ vs. (Ag/AgCl) [38].

Lee et al. has researched a nicotine electrochemical sensor where a copper hexacyanoferrate-polypyrrole (CuHCF–PPy) nanocomposite was deposited directly onto reduced graphene oxide (rGO) by a direct self-assembly technique. From the impedance results, they obtained two semicircles in their Nyquist plot. This was expected and is due to the presence of two layers’ of materials comprising of rGO and the metal layer (CuHCF) or metal-polymer layer (CuHCF-PPy) on the electrodes. Therefore, from the Bode plot, two phase angles were observed. The phase angle in the high (*f*1) frequency regions was attributed to the *R*_ct_ which happens across the electrode-electrolyte interface (CuHCF or CuHCF-PPy/solution). Meanwhile, the second phase angle (*f*2) was due to the CuHCF or CuHCF-PPy/rGO interface [36]. In research from a different perspective, Ratautaite et al. used Bode plots to evaluate the best frequency for further evaluation of capacity changes as a result of theophylline addition. They found that most sensitive impedance changes were in the frequency range from 10 Hz to 100 Hz. Therefore, in that frequency range, the capacitance changes at certain frequencies were further evaluated [37]. On the other hand, Al-Mokaram et al. used a Bode plot to study the frequency region of *R*_ct_ of modified electrode nanocomposite films consisting of polypyrrole-chitosan-titanium dioxide (Ppy-CS-TiO_2_) in the development of a non-enzymatic glucose biosensor. It was found to collect in the frequency range of 0.01–10,000 Hz. The shifting of peaks toward the low frequency region of 1–0.01 Hz for composite and nanocomposite electrodes indicates the fast electron-transfer behavior of the nanocomposites (Figure 6). A perfect linear portion was observed at lower frequencies for the nanocomposite electrode compared to other electrodes. The results indicated that the Ppy-CS-TiO_2_ nanocomposite was successfully designed and it facilitated a diffusion-limited process at the electrode-solution interface [38].

### 2.3. Dielectric Constant (ε_r_)

Impedance measurements analysis can also provide data on the intrinsic dielectric constant and dielectric loss properties of an electrochemical sensor [39]. Dielectric studies are used to understand the mechanism of conduction and dielectric properties that may be valuable in developing a device’s performance and in the design of the electronic devices. According to Ramesan and Santhi, who studied conducting polymer composites of polypyrrole (PPy) with different silver doped nickel oxide (Ag–NiO) nanocomposites, the dielectric constant was found to depend on the polarizability of dipoles in the direction of the applied field. From their results, they found that PPy exhibited a lower dielectric constant compared to all of the nanocomposites they studied [40]. Their argument was supported by Anilkumar et al., who explained that the lower dielectric constant seen in conducting polymers may be attributed to the interfacial polarization of the composite materials. Interfacial polarization mainly arises from the electrical heterogeneities of the composite materials [41]. Strong interfacial interactions between the polymer and nanoparticles reduce the macromolecular chain’s cohesive forces, which increases the dielectric constant of the composite. With further loading of the composites (>10 wt%) in the PPy matrix, it was found to decrease the dielectric constant. This is thought to be due to the formation of aggregates within the PPy matrix. They also found that the dielectric loss of composites is higher than that of PPy. The higher dielectric loss observed might be due to the high surface area, surface domain polarization and the quality of the electrical network formation [42]. With further loading of the composite, the dielectric loss was found to decrease. This may be due to the formation of clusters or discrete aggregates in the PPy matrix, which can prevent the migration of charge carriers through the polymer. 

### 2.4. Dielectric Loss (tan δ)

Dielectric loss is represented by the dissipation factor (tan *δ*) which is the amount of dissipated energy or electrical loss by insulating material when a voltage is applied to the material [40]. The dielectric loss of a material is directly related to the electrical conductivity of the corresponding matrix. Usually, from the graph of dielectric loss, it always decreases as the frequencies are increased. This is attributed to the time lagging associated with the orientation of dipoles within the polymer matrix. Ramesan and Santhi also studied dielectric loss in their conducting polymer composite of polypyrrole (PPy) with different silver doped nickel oxide (Ag–NiO) content nanocomposite system and found that the tan *δ* of composites was higher than that of PPy alone. This was similar to their findings in the dielectric constant. Furthermore, when they increased the nanoparticle content, the tan *δ* value of the composite also increased. This may be due to the high surface area, surface domain polarization, and the quality of the electrical network formation. With further loading of nanocomposites (>10 wt%), the tan *δ* value was seen to decrease. This might be caused by the formation of clusters or discrete aggregates in the PPy matrix, which then prevented the migration of charge carriers through the polymer [40]. Besides that, Ramesan and co-workers also worked on different types of nanocomposite and polymer matrices. They studied poly (vinyl cinnamate) (PVCin) with different ratios of zinc oxide (ZnO). Their results showed a similar pattern to the other studies [43].

### 2.5. Impedimetric PPy Based Electrochemical Sensors and Biosensors

In most EIS studies that involve electrochemical sensors, the use of an electroactive probe/indicator has been employed to evaluate an electrode’s signal activity. The advantage of using an electroactive probe is that it can serve as a reference point for impedance studies. For example, Arabali et al. fabricated an amplified tramadol electrochemical sensor based on the surface modification of pencil graphite electrode (PGE) by CuO nanoparticle (CuO-NPs) and polypyrrole (PPy). From the impedance results obtained, it confirmed the modification of PGE with PPy and PPy + CuO-NPs was able to improve the electrical conductivity of the sensor and exhibited a highly sensitive electroanalytical sensor for the determination of tramadol [44]. Furthermore, in humidity sensor applications, impedance analysis is commonly used to study the effect on a conducting polymer composite with various different compositions on the percentage of relative humidity measurement, RH%. Su et al. reported that the addition of PPy-Ag into pristine SnO_2_ helped to reduce the impedance resistance, thereby increasing its sensitivity especially at low RH%. The study also proved that the impedance decreased as the amount of PPy-Ag increased. The addition of PPy-Ag helped to increase the mobility of solvated ions inside the system [45]. Meanwhile, Jlassi et al. reported that their findings did not follow this trend. They studied the effect of a combination PPy-Ag with modified halloysite nanoclay (HNT) films on RH% sensitivity at three different wt.% (0.25 wt.%, 0.5 wt.%, and 1 wt.%) amounts. The best finding was achieved using PPy-Ag 0.5 wt.% film due to its hydrophilic behavior. They found that the impedance decreased as the humidity level increased. This is because the higher the humidity level, the higher the amount of moisture absorbed onto the PPy-Ag film, thereby increasing the conductivity of the film [46]. 

## 3. Voltametric Sensing Mode

Voltametric sensing mode is a measurement technique where a current is produced by sweeping the potential applied between a reference electrode and a conducting polymer or a conducting polymer modified electrode over a range that is associated with the redox reaction of the analyte [47,48]. It is also known as voltammetry analysis. There are a number of voltammetry techniques used which are described below.

### 3.1. Cyclic Voltammetry (CV)

**Figure 7:**
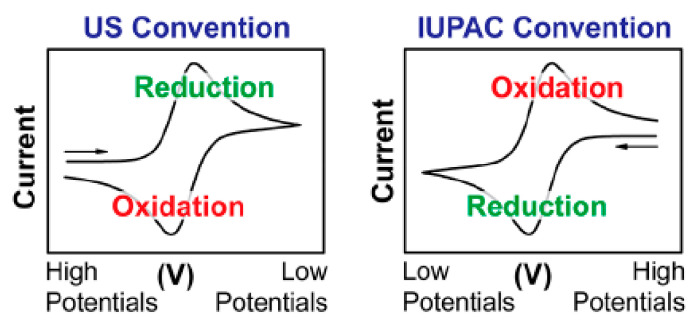
Examples of “duck” shaped cyclic voltammograms [49]. Reproduced with permission from [N. Elgrishi], [A Practical Beginner’s Guide to Cyclic Voltammetry]; published by [ACS Publications], [2017].

One of the most powerful and popular voltammetry techniques used to investigate the reduction and oxidation processes of molecular species is cyclic voltammetry (CV). CV is an analysis to study electron transfer-initiated chemical reactions, which includes catalysis. Generally, a typical graph with a “duck” shaped curve will be obtained from CV analysis which is called a voltammogram or a cyclic voltammogram, as shown in Figure 7.

From the graph, it can be seen that the *x*-axis represents the applied potential (*E*) that is imposed on the system; meanwhile, the *y*-axis is the resulting current (*i*) passed, which is the response during the measurement. Some direct information can be obtained from the CV graph, such as that at the potential axis (*x*-axis), it contains an arrow which indicates the direction of the scanned potential used to record the data. Besides that, it also indicates the beginning and sweep direction of the first segment (or “forward scan”). Sometimes, a crucial parameter also can be found in the graph which is scan rate (*υ*). It indicates that the potential was varied linearly at the speed (scan rate) during the experiment: for example, *υ* = 100 mV/s [49].

**Figure 8:**
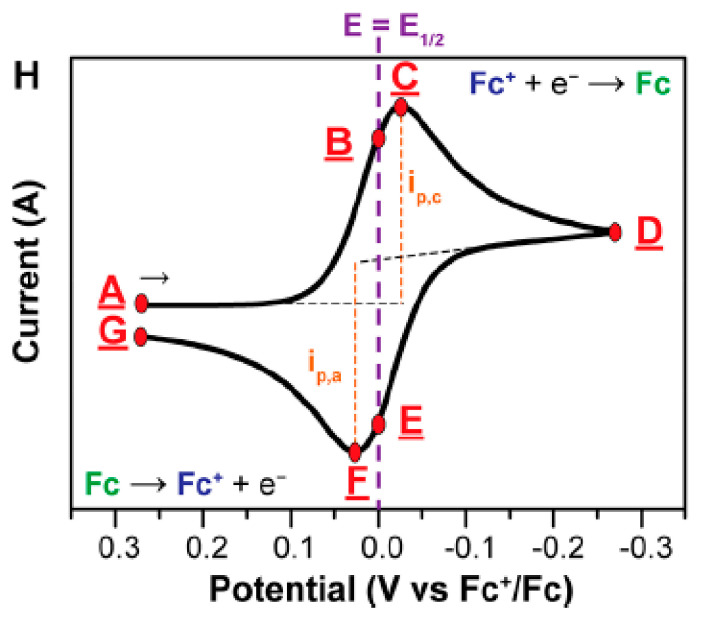
Concentration profiles (mM) for Fc^+^ (blue) and Fc (green) as a function of the distance from the electrode (D, from the electrode surface to the bulk solution, e.g., 0.5 mm) at various points during the voltammogram analysis [49]. (Current flow from A to G.) Reproduced with permission from [N. Elgrishi], [A Practical Beginner’s Guide to Cyclic Voltammetry]; published by [ACS Publications], [2017].

Figure 8 shows the “duck”-shaped voltammogram of a reversible reduction of 1 mM Fc^+^ solution to Fc, at a scan rate of 100 mV/s. As the potential is scanned negatively (cathodically) from point A to point D, (Fc^+^) is reduced to Fc and it is steadily depleted near the electrode. Simultaneously, a peak cathodic current (*i*_p,c_) can be observed at point C [50]. It is dictated by the delivery of additional Fc^+^ via diffusion from the bulk solution. The volume of solution at the surface of the electrode containing the reduced Fc, called the diffusion layer, continues to grow throughout the scan. This will then slow down the mass transport of Fc^+^ to the electrode. Thus, upon scanning of more negative potentials, the diffusion rate of Fc^+^ from the bulk solution to the electrode surface becomes slower, resulting in a decrease in the current as the scan continues (C→D). When it reaches the switching potential, D, the scan direction is reversed, and the potential is scanned in the positive (anodic) direction. While the concentration of Fc^+^ at the electrode surface is depleted, the concentration of Fc at the electrode surface is increased, satisfying the Nernst equation. The Nernst equation (Equation (4)) can be used in order to predict how a system will respond to a change of concentration of species in solution or a change in the electrode potential. The Fc presented at the electrode surface is oxidized back to Fc^+^ as the applied potential becomes more positive. At points B and E, the concentrations of Fc^+^ and Fc at the electrode surface are equal, following the Nernst equation, *E* = *E*_1/2_. This corresponds to the halfway potential between the two observed peaks (C and F) and provides a straightforward way to estimate the *E*^0′^ for a reversible electron transfer, as noted above. The two peaks are separated due to the diffusion of the analyte to and from the electrode.

(4) $$E=E^{0}+\frac{RT}{nF}ln\frac{\left(Ox\right)}{\left(Red\right)}=E^{0}+2.3026\frac{RT}{nF}log_{10}\frac{\left(Ox\right)}{\left(Red\right)}$$

Generally, in an electrochemical sensor, cyclic voltammetry is used to study the effect of a conducting polymer’s modification towards its current intensity peak. Previously, Kwak et al. reported on the modification of PPy-base with carbon doped polydimethylsiloxane (PPy/ CPDMS). Their results showed current peaks during the reduction and oxidation exhibited at a voltage nearby 1.5 V and −1 V, respectively [51]. As the scan rate increases, the currents peak magnitude tends to increase due to the higher scan rate facilitating a thin diffusion layer between the electrolyte and the PPy surface [49]. However, as the scan rate was increased, the voltage at the corresponding current peaks were not identical during the redox reaction. This implied some degree of chemical irreversibility possibly caused by insufficient electron transfer because of the fast scan rate, or the decomposition of the PPy surface [52,53]. 

Zaabal et.al modified a glassy carbon electrode with polypyrrole (PPy/GCE) to be used as a promising electrode for electrochemical sensing of adefovir (ADV). They reported a weak anodic peak current obtained at 1.559 V for the unmodified electrode. By modifying the GCE with PPy, the anodic peak was shifted to a more negative potential which was 1.484 V accompanied by an enhancement in the peak height of ADV. The higher anodic response of ADV at the PPy/GCE electrode showed that this modified electrode was more sensitive than GCE alone. The enhanced signals and shift of potential peak towards the negative direction indicated that the modified electrode improves electrochemical reactivity of ADV oxidation as compared to bare GCE. This was probably mainly due to the large effective surface area and subtle electronic conductivity of PPy film, which was beneficial to promoting the electron transfer reaction [54].

Besides having a shift in potential axes, the changes in current peak also give important information; for example, in research done by Chen et al. [55] where they prepared a novel polypyrrole/glassy carbon electrode (PPy/GCE) core-zeolitic imidazolate framework-8 (ZIF-8) shell structure composite for quercetin (QR) determination. They found that the current peak of the QR sensor composed of ZIF-8/PPy/GCE was higher than the bare PPy/GCE electrode. It was due to a larger electrocatalytic surface obtained from ZIF-8 and high charge collectability of the host PPy [54]. A similar trend was observed by Hu at el. [56], where they prepared a novel electrochemical sensor based on ion imprinted polypyrrole and reduced graphene oxide (PPy/rGO) composite for trace level determination of cadmium ion (Cd(II)) in water. They found that with the addition of rGO into PPy/GCE, it increased the rate of electron transfer on the electrode surface and amplified the signal response [56].

Furthermore, Yu et al. developed a new electrochemical sensor based on titanium dioxide (TiO_2_) and a PPy molecularly imprinted polymer (MIP) nanocomposite for the highly selective detection of p-nonylphenol in food samples. On just the bare GCE, a well-defined reversible redox peak could be observed. When the GCE was modified with PPy and TiO_2_, the current intensity peak was obviously enhanced. It suggested that the modification could result in a larger electrochemical surface area, due to the cavities found in the PPy matrix which could accelerate electron transfer of (Fe(CN)_6_)^3−/4−^. After incubation with p-nonylphenol, the MIP absorbed p-nonylphenol molecules and blocked the cavities in the PPy matrix. Thus, the redox peak current intensity decreased as a result of the limitations of electron transfer. In contrast, the electrode modified with PPy and nanoimprinted TiO_2_ exhibited a lower current intensity peak compared to PPy with TiO_2_ MIP [57]. 

However, this was found to be different from the findings Ma et al., where they developed an electrochemical biosensor based on sodium alginate-polypyrrole/Au nanoparticles (SA-PPy/AuNPs) nanocomposite for the detection of miRNAs [58]. They reported that the current peak decreased after the modification of bare GCE. The redox peak current of Fe(CN)_6_^4−/3−^ slightly decreased due to the poor conductivity of SA and modified hair pin (H1). This could slow down the electron transfer on the surface of the electrode. The GCE/SA-PPy/AuNPs/H1 modified with miRNA-21 and modified hair pin (H2) formed a large number of double helix DNA structures on the surface of the electrode due to the occurrence of the CHA reaction, with a reduction in the redox peak current of Fe(CN)_6_ ^4−/3−^. Finally, a slight decrease in redox peak current was observed after the copper ion (Cu(II)) complex was inserted onto the double helix DNA structure. This could be attributed to the dissolution of the Cu(II) complex in the mixture of dimethyl sulfoxide (DMSO) and water (H_2_O) (volume ratio 7:3). However, the observed poor solubility may have triggered a blockage of the electron transfer between the surface of the electrode and the electrolyte [58].

Besides conducting polymer modifications, the current peak of CV can also be affected by the concentration of a sample [30,59,60,61]. Zhang et al. designed and constructed an electrochemical ammonia sensor based on Ni foam-supported silver/polypyrrole and platinum nanoparticles electrode (Pt-Ag/PPy-NiF). They studied the effect of ammonia concentration on the oxidation current peak of the PPy/Pt/Ag/NiF electrode and found that its current peak increased when the ammonia concentration increases. This happened because of the strength of the synergistic effect between Ni foam and Pt nanoparticles [59]. Suvina et al. developed a polypyrrole-reduced graphene oxide hydrogel composite electrode for the detection of metal ions. They investigated the effect of metal ion concentration on the PPy-rGO hydrogel electrode and observed that the formation of a multilayer metal ion complex accumulated as a pre-deposited monolayer helped increase the peak current [61]. Meanwhile, Devi et al. prepared a mixture of PPy with synthesized zinc oxide nanoparticles (ZnO-NPs) which were then electropolymerized onto a platinum (Pt) electrode to form a ZnO-NPs–polypyrrole (PPy) composite film. Then, xanthine oxidase (XOD) was immobilized onto the nanocomposite film through physiosorption to study the effect of XOD concentration on the ZnO-NPs/PPy/Pt electrode. They reported that the increases in oxidation current was due to the increased concentration of hydrogen peroxide (H_2_O_2_) produced during enzymatic reaction [30]. However, it is in contrast to Alagappan et al.’s study, which prepared an electrochemical cholesterol biosensor based on the cholesterol oxidase (ChOx) enzyme immobilized on a gold nanoparticle—functionalized -multiwalled carbon nanotube (MWCNT)—polypyrrole (PPy) nanocomposite modified electrode. They reported that the anodic and cathodic peak currents decreased with an increase in cholesterol concentration. This happened because of an absence of a redox mediator in the system which reduced the electron hopping from the analyte to the enzyme modified electrode [60].

Besides that, the potential difference between the anodic and the cathodic peaks also can be extracted from a cyclic voltammogram. Lo et al. prepared a PPy/CNT/NH_2_-ITO composite by electropolymerization onto polypyrrole-aminophenyl-modified flexible indium tin oxide (PPy/NH_2_/ITO) electrodes coated with multi-walled carbon nanotubes (CNTs), in the presence of ethylene glycol-bis(2-aminoethylether)-tetraacetic acid (EGTA) as a chelator. They reported that the potential difference of PPy films deposited onto bare ITO was 430 mV [62]. This was high compared to bare ITO which only exhibited 165 mV [63]. This difference can be linked to the absence of any adhesion between the PPy layer and the bare ITO surface. Meanwhile, in the case of PPy/NH_2_-ITO, the presence of NH_2_ on ITO contributed to an increase in electronic transfers leading to a lower ΔE (181 mV). For PPy/CNT/NH_2_-ITO, ΔE = 321 mV. The CVs were consistent with those obtained by impedance measurement. 

Pineda et al. [64] investigated the effect of polymerization time on the potential and current peak on polypyrrole (PPy) films with a micro tubular structure decorated with gold nanoparticles. The result showed that the anodic current peak from the voltammogram of the PPy film exhibited a cauliflower-like structure, occurring at 0.05 V, and there was found a small cathodic peak at −0.8 V that was led by a small hump at −0.5 V. Furthermore, these anodic and cathodic current peaks were well-defined at ca. 0.28 V and −0.45 V, respectively, as the electropolymerisation time was increased and the tubular structure was formed. This shows that the tubular structure exhibited a better separation between the faradaic and capacitive contributions in a polymeric deposited film [64]. 

Another aspect that can be studied through cyclic voltammogram is electrocatalytic behavizr [65,66]. Xing et al. studied the electrocatalytic behavior of polypyrrole/platinum (PPy/Pt) nanocomposites toward hydrogen peroxide (H_2_O_2_) reduction. They found that in the absence of H_2_O_2_, no reduction peak was observed with bare glassy carbon electrode (GCE), PPy/GCE, and PPy/Pt/GCE. Upon the addition of H_2_O_2_, no obvious current from the reduction of H_2_O_2_ was observed at bare GCE other than a minor increase in the background current. While only a weak reduction peak for H_2_O_2_ at about −0.28 V was observed on the PPy/GCE electrode, in contrast, on the PPy/Pt/GCE electrode, there was a remarkable reduction peak of H_2_O_2_ obtained of around −0.2V. This was even higher than the bare Pt electrode in terms of reduction peak current value, indicating that the PPy/Pt/GCE might provide a better electrocatalytic effect than the bare Pt electrode [65]. 

The electrocatalytic oxidation of an adenine and guanine mixture at bare and modified PPy/graphene/GCE electrodes were studied by Gao et al. [66]. They reported that there was no oxidation signal observed in the CV curves of the PPy/graphene/GCE electrode due to a blocking effect but there was a high current peak observed with the modified PPy/graphene/GCE electrode. The report also concluded that the overoxidized polypyrrole/graphene/glassy carbon electrode (PPyox/graphene/GCE) electrode had the highest current peak of adenine and guanine oxidation which indicated the highest electrocatalytic activity [66].

### 3.2. Limit of Detection (LOD)

Voltammetry can be used to obtain quantitative information which is deduced from peak current intensity [48]. Usually, it is used to determine the sensitivity value of the sensor. Sensitivity, analytical sensitivity, functional sensitivity, lower limit of detection (LOD), etc., are terms used to describe the smallest concentration of a measure that can be measured (detected) with statistical significance employing a given analytical procedure [67]. It is also defined as the minimum input quantity that can be distinguished with more than 99% reliability [68]. *LOD* is also a valuable quantitative measurement usually in the healthcare industry where it can be used as a biomarker in the detection of disease, environmental pollutants such as heavy metals, and other chemical contaminants that are part of the environmental liability in contemporary societies [69]. The value of the *LOD* can be determined through calculation using Equation (5). 

**Figure 9:**
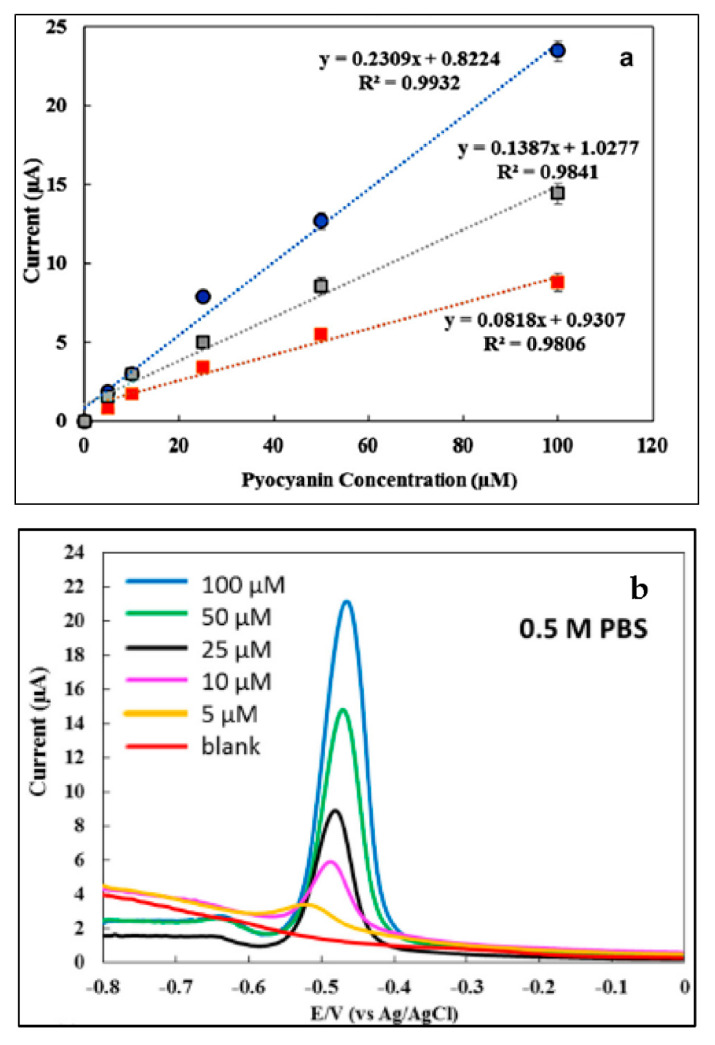
Example graphs of current response with different concentrations of pyocyanin biomarker using a DPV analyzer where the calibration curve for the three different mediums (0.5 M PBS at pH 7.4 (blue), human urine (grey) and human saliva (red)) at the potential range of −0.8 V to 0 V (**a**) and DPV current response of pyocyanin (5–100 μM) in 0.5 M PBS (**b**) [70]. Reproduced with permission from [J.I.A.Rashid], [An electrochemical sensor based on gold nanoparticles-functionalized reduced graphene oxide screen printed electrode for the detection of pyocyanin biomarker in Pseudomonas aeruginosa infection]; published by [Elsevier], [2020].

The equation to calculate the *LOD* is:

(5) $$LOD=\frac{3\times SD}{m}$$

where *SD* is the magnitude of the error bar at blank while *m* is the slope of the calibration curve of the blank [69]. Before it can be calculated, a graph of current or current density with different concentrations of the sensing material needs to be plotted (Figure 9a).

The current peak (*i*_p_) value can be obtained either from the peak of a cyclic voltammogram (CV), differential pulse voltammogram (DPV), or square wave voltammogram (SWV). Most researchers use DPV and SWV to determine the current peak (Figure 9b). This is because of the high sensitivity of the technique compared to CV. In general, CV can provide essential information, such as the reversibility process and types of redox processes present in an analysis (matrix, analyte, and electrode); however, DPV and SWV are used for quantitative determinations [71]. 

### 3.3. Differential Pulse Voltammogram (DPV)

**Figure 10:**
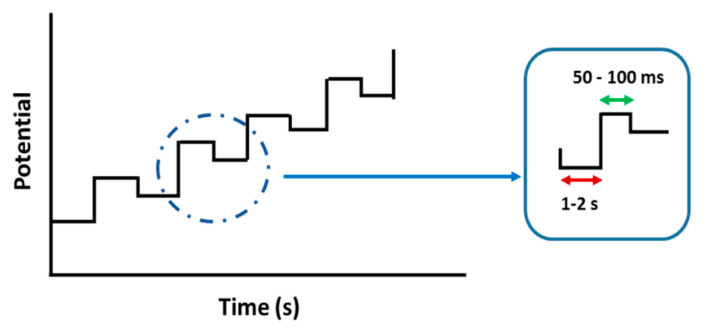
Diagram of the application of pulses in differential pulse voltammetry (DPV).

DPV is a technique that involves applying amplitude potential pulses on a linear ramp potential. In DPV, a base potential value is chosen at which there is no faradaic reaction and is then applied to the electrode. The base potential is increased between pulses with equal increments. The current is immediately measured before the pulse application and at the end of the pulse, and the difference between them is recorded (Figure 10). DPV is similar to the first derivative of a linear voltammogram in which the formation of a peak is observed for a given redox process. In the linear sweep technique, it has a shape similar to a wave, and the first derivative originates a peak. 

As in polarography (dropping mercury electrode), the qualitative information of an analyte is given by its half-wave potential (E_1/2_), which corresponds to the potential at half the wave height. Similarly, in DPV, the peak potential, E_p_, can be approximately identified through E_1/2_. Increasing the irreversibility, E_p_ deviates from E_1/2_ as the base of the peak widens and its height decreases. The DPV is therefore a graph of differences between measured currents and applied potentials as shown in Figure 9b [69,72].

### 3.4. Square Wave Voltammogram (SWV)

Meanwhile, SWV is the fastest and the most sensitive pulse voltammetry technique. The detection limits can be compared with those of chromatographic and spectroscopic techniques. In addition, the analysis of the characteristic parameters of this technique also enables the evaluation of the kinetics and mechanism of the electrode process under study [71,73]. According to Marie et al., the lower limit of detection value means the lowest concentration of glucose (analyte) that can be detected by the device [74]. 

Zhang et al. constructed an aqueous ammonia sensor using a low cost, high sensitivity, and great stability construct based on Ni foam-supported silver/polypyrrole and platinum nanoparticles electrode (Pt-Ag/PPy-NiF). They found that the ammonia sensor showed a sensitivity of 0.089 mA μM^−1^ with a detection limit of 37 nM and a wide linear range, as well as possessing a good anti-interference capability against many common ions found in water. The Pt-Ag/PPy-NiF electrode possessed a lower detection limit and higher sensitivity compared to most electrochemical aqueous ammonia sensors. Its excellent performance can be mainly attributed to the large specific surface area of the electrode and great electrocatalytic ability of Pt nanoparticles [59]. 

Salunke et al. prepared a sensor device by electrodepositing gold nanoparticles onto a single PPy nanowire for arsenic detection. They found that the sensitivity of Au-NPs decorated Ppy-NW towards arsenic (for two set of as conc. range) was 0.22 and 4.38 µM^−1^ with a good detection limit of 0.32 μM. Their result was better in respect of sensitivity and LOD, and this could be due to the change in sensor dimensions where they used nanowire as a base material and the gold nanoparticles as the sensing material. This led to the finding of a high surface area as being a dimension factor for the enhancement of sensor sensitivity with rapid detection and accuracy [75]. 

In other work done by Zaabal et al., they studied a polypyrrole modified glassy carbon electrode (PPy/GCE) for the electrochemical sensing of adefovir (ADV). ADV is a broad-spectrum antiviral agent whose action is as a DNA polymerase inhibitor. The detection limit of ADV obtained was 3.10 nM. Moreover, they also determined ADV in human serum and urine and found that the detection limit was 0.06 μM and 0.04 μM, respectively. Their detection limit value was much lower compared to the findings in other research works [76,77]. This showed that PPy/GCE can be an effective alternative sensor for the electrochemical determination of ADV in commercial pharmaceutical dosage forms and biological fluids. 

### 3.5. PPy Based Voltammetric Sensor

In order to develop sensitive detection instruments, different signal amplification strategies also can be utilized to improve the sensitivity of the sensors used. Ma et al. designed an ultrasensitive and specific electrochemical biosensor for the determination of miRNAs based on SA-PPy/AuNPs. It was analyzed using the SWV technique with a dual signal amplification strategy involving the catalytic hairpin assembly (CHA) reaction and Cu^2+^/Fe^3+^ catalytic reaction [58]. They found that, with the usage of this dual signal amplification strategy and a sensitive analyzer technique, which was SWV, the LOD value was significantly improved and could be as low as 0.34 fM compared to other research work [78,79,80,81]. Table 1 shows the comparison of different methods and signal amplification strategies with miRNA-21 as the target. The CHA reaction was triggered and numerous double helix-DNA were formed in the presence of the target miRNA. Furthermore, the intercalated planar Cu (II) complex could bind with the double-helix DNA, which was employed as a signal amplification molecule in the presence of Fe^3+^. After the dual signal amplification, the reduction of the Cu (II) complex was recorded to represent miRNA levels. 

Table 2 summarizes the electrochemical sensing modes of PPy based electrochemical sensors reviewed in this paper with their possible applications.

## 4. Conclusions

This review presents an overview of the two main electrochemical sensing modes used with electrochemical sensors based on PPy conducting polymers and outlines the significant advances in this field. In this review, the PPy heterocyclic-based conducting polymer was chosen due to its good environmental stability, high electronic conductivity, and biocompatibility. Conducting polymers are considered good sensitive materials for the development of selective, specific, and stable sensing devices. However, having PPy alone as the conducting polymer might not give the best results for an electrochemical sensor or biosensor application. Therefore, modification of the conducting polymer is the way to enhance its properties. To get an in-depth overview of how the modification can improve electrochemical and biosensors, the two main electrochemical techniques, impedimetric and voltametric, should be utilized. From this review, it can be seen that these two techniques play an important role in order to determine the resistivity, reactivity, and sensitivity of the PPy based conducting polymers for use in electrochemical sensors and biosensors. Besides that, they can also help the understanding of the reactivity that occurs when modification of the PPy conducting polymer has been done.

## Figures and Tables

**Table 1:** Comparison of different methods and signal amplification strategies with miRNA-21 as the target.

Detection Method	Signal Amplification Strategy	LOD Value	References
Fluorescence	Ligase mediated amplification	314 fM	Chan et al. [80]
DPV	Fe(CN)_6_^3+^	10 fM	Low et al. [79]
Chemiluminescence	Cascade enzyme catalytic reaction	1 fM	He et al. [81]
DPV	CHA reaction and RCA	13.5 fM	Wang et al. [78]
SWV	CHA reaction and Cu^2+^/Fe^3+^ catalytic reaction	0.34 fM	Ma et al. [58]

**Table 2:** Summary of electrochemical sensing modes of PPy-based electrochemical sensors with their applications.

Material	Modification/Improvement	Electrochemical Sensing Mode	Application	References
-	SnO_2_	Impedimetric	Humidity sensor	Su et al. 2020 [45]
PPy	Ag-SnO_2_			
PPy	Ag (0.5 wt.%)-HNT-DMA	Impedimetric	Humidity sensor	Jlassi et al. 2020 [46]
PPy	ITO	Impedimetric Voltammetric	Lead ion detector	Lo et al. 2020 [62]
PPy	NH_2_-ITO			
PPy	CNT-NH_2_-ITO			
PPy	MIP(UA)	Impedimetric	Electrochemical quartz crystal microbalance-based sensor	Plausinaitis et al. 2020 [82]
	GCE	Impedimetric Voltammetric	Dectector for miRNA-21	Ma et al. 2020 [58]
PPy	GCE-SA-AuNPs			
	CFP	Impedimetric Voltammetric	MMA detector	Akshaya et al. 2020 [31]
PPy	CFP			
PPy	CFP-PdAu			
PPy	GCE	Voltammetric	Electrochemical sensing of Adefovir	Zaabal et al. 2020 [54]
PPy	Pt-Ag-NiF	Voltammetric	Aqueous ammonia sensor	Zhang et al. 2020 [59]
PPy	Carbon doped polydimethylsiloxane	Impedimetric Voltammetric	Resistive sensor	Kwak et al. 2019 [51]
	GCE	Impedimetric Voltammetric	Food sensor: p-nonylphenol analysis (milk powder)	Yu et al. 2019 [57]
PPy	TiO_2_-MIP-Nafion-GCE			
PPy	TiO_2_-MIP-Nafion-GCE in 0.1mM p-nonylphenol			
PPy	NP-TiO_2_-MIP-Nafion-GCE			
PPy	TiO_2_ NIP/Nafion/GCE			
	CuHCF	Impedimetric voltammetric	Nicotine detector	Lee et al. 2019 [36]
PPy	CuHCF			
	rGO			
	rGO-CuHCF			
PPy	rGO-CuHCF			
PPy	SS-Au	Impedimetric voltammetric	Detection of hydroxylamine, nitrite and their mixture	Pineda et al. 2018 [64]
PPy	GCE	Impedimetric voltammetric	Quercetin detector	Chen et al. 2019 [55]
	ZIF-8 nanoparticle			
PPy	ZIF-8 nanoparticle			
PPy	rGO-GCE	Voltammetric	Cadmium detector	Hu et al. 2019 [56]
	GCE	Impedimetric Voltammetric	Biosensor: microRNA detector	Bao et al. 2019 [32]
PPy	Au-rGO-GCE			
PPy	MCM-41	Impedimetric	Humidity sensor	Qi et al. 2018 [83]
	GCE	Impedimetric Voltammetric	Biosensor for cholesterol detection	Alagappan et al. 2018 [60]
PPy	GCE			
PPy	GCE- Au-*f*-MWCNT			
PPy	Au-*f*-MWCNT-ChO*x*-GCE			
PPy	Cu_2_O-ITO	Impedimetric	Imprinted PEC sensor	Chen et al. 2018 [31]
PPy	NIP-Cu_2_O-ITO			
PPy	MIP-Cu_2_O-ITO			
PPy	rGO hydrogel	Voltammetric	Metal ions sensor	Suvina et al. 2018 [61]
Ppy	Au-NPs-NW	Voltammetric	Arsenic detector	Salunke et al. 2017 [75]
PPy	Pt nanocomposite	Voltammetric	Non-enzymatic electrochemical sensor	Xing et al. 2015 [65]
PPy	Pt-GCE			
PPyox	Gr nanocomposite-GCE	Voltammetric	DNA & RNA sensor: detection of adenine and guanine	Gao et al. 2014 [66]
	Pt	Impedimetric Voltammetric	Xanthine biosensor	Devi et al. 2011 [30]
PPy	Pt			
PPy	ZnO-NPs-Pt			
PPy	XOD-ZnO-NPs-Pt			
